# Relevance of the light signaling machinery for cellulase expression in *trichoderma reesei *(*hypocrea jecorina*)

**DOI:** 10.1186/1756-0500-3-330

**Published:** 2010-12-07

**Authors:** Miklós Gyalai-Korpos, Gáspár Nagy, Zoltán Mareczky, André Schuster, Kati Réczey, Monika Schmoll

**Affiliations:** 1 Budapest University of Technology and Economics, Department of Applied Biotechnology and Food Science, 1111 Budapest Szent Gellért tér 4., Hungary; 2 Vienna University of Technology, Research Area Gene Technology and Applied Biochemistry, 1060 Wien, Gumpendorfer Strasse 1a/1665, Austria

## Abstract

**Background:**

In nature, light is one of the most important environmental cues that fungi perceive and interpret. It is known not only to influence growth and conidiation, but also cellulase gene expression. We therefore studied the relevance of the main components of the light perception machinery of *Trichoderma reesei *(*Hypocrea jecorina*), ENV1, BLR1 and BLR2, for production of plant cell wall degrading enzymes in fermentations aimed at efficient biosynthesis of enzyme mixtures for biofuel production.

**Findings:**

Our results indicate that despite cultivation in mostly dark conditions, all three components show an influence on cellulase expression. While we found the performance of the enzyme mixture secreted by a deletion mutant in *env1 *to be enhanced, the higher cellulolytic activity observed for *Δblr2 *is mainly due to an increased secretion capacity of this strain. *Δblr1 *showed enhanced biomass accumulation, but due to its obviously lower secretion capacity still was the least efficient strain in this study.

**Conclusions:**

We conclude that with respect to regulation of plant cell wall degrading enzymes, the blue light regulator proteins are unlikely to act as a complex. Their regulatory influence on cellulase biosynthesis involves an alteration of protein secretion, which may be due to adjustment of transcription or posttranscriptional regulation of upstream factors. In contrast, the regulatory function of ENV1 seems to involve adjustment of enzyme proportions to environmental conditions.

## Findings

The ascomycete *Trichoderma reesei *(anamorph of *Hypocrea jecorina*) is one of the most prolific cellulase producing microorganisms, its efficient enzyme mixture being used in several processes of textile, food and pulp and paper industries [1-3]. Moreover a new market potential is arising with the commercialization of cellulosic ethanol plants: however, a main bottleneck for the economic success of the production of the second generation biofuels is the price of cellulolytic enzymes [4]. Strain improvement in *T*. *reesei *for plant cell wall degrading enzyme production can become more efficient with the use of the genome sequence [5,6]. Interestingly, analysis of the genome of *T. reesei *revealed an unexpectedly low number of genes encoding cellulolytic enzymes - despite the high efficiency of the cellulase mixture produced by this fungus. Besides improving the produced enzymes themselves or the efficiency of the promotors by which their expression is controlled, one strategy to elucidate the underlying mechanisms responsible for this high efficiency of *T. reesei *can be the investigation and exploitation of signal transduction processes [7,8] during growth on cellulosic substrates. Signaling mechanisms greatly contribute to successful adaptation and survival by receiving and interpreting numerous biotic and abiotic factors one of which is light. In contrast to plants, which utilize light as energy, for fungi light is merely a source of information. Blue light affects or initiates a number of physiological processes in fungi in general and also in *Trichoderma*, e.g. growth, conidiation and numerous metabolic pathways [9,10]. Many effects of light are common within the fungal kingdom and also the pathways of light sensing and its elements often share significant homology [11].

The photobiology of *Trichoderma *spp. has been investigated in considerable depth for decades [12]. Orthologues of the well studied *Neurospora crassa *photoreceptor genes *wc-1 *and *wc-2 *[13] genes were described in *Trichoderma atroviride *[14] and subsequently also in *T. reesei *[15]. The *T. reesei *blue light regulators (BLR1 and BLR2) have similar structural domains (PAS/LOV) and light independent regulatory roles were also reported for these proteins [15]. In *T. atroviride *also BLR independent light sensing routes have been proposed [16]. ENVOY, a PAS/LOV domain protein in *T. reesei*, which shares similarity with the *N. crassa *photoreceptor VIVID [17-19] is crucial in light tolerance and modulates cellulase transcription in a light dependent manner [20]. Recently, also an influence of the two photoreceptors BLR1 and BLR2 on cellulase gene transcription has been shown [15] suggesting that these regulators act positively on this process. In *Trichoderma*, also cAMP levels are responsive to light [21] and cAMP is involved in regulation of cellulase levels [22], which indicates an action via phosphorylation of transcription factors by cAMP dependent protein kinase A. Moreover, two G-protein alpha subunits (GNA1 and GNA3, which impacts cAMP levels) have been shown to exert a considerable light dependent influence on transcription of the major cellulase gene *cbh1/cel7a *[23,24]. However, since these high levels of transcription did not result in an equally high production capacity of the respective mutant strains (M. Schmoll, unpublished results), further (presumably light-dependent) regulatory checkpoints at the level of translation and/or secretion can be expected.

Based on these findings we assumed that the major components of the light response pathway (BLR1, BLR2 and ENV1) could be crucial regulators or checkpoints in (light dependent) production of extracellular enzymes. Therefore we aimed to investigate the relevance of the light signaling machinery, which obviously plays an important role in cellulase regulation, for industrial fermentations. Analysis of strains defective in light sensing showed that the blue light regulatory proteins BLR1, BLR2 and ENV1 are indeed involved in regulation of expression and secretion of plant cell wall degrading enzymes, even in the predominantly dark conditions of a biotechnological steel fermentor. Consequently, our study revealed new targets for improvements of cellulase production by modification of the light signaling machinery.

### Presence of promotor motifs associated with light response in genes encoding plant cell wall degrading enzymes

Because of the reported influence of light as well as of ENVOY, BLR1 and BLR2 on regulation of cellulase expression [15,20] a direct regulation of cellulolytic genes by the transcription factors BLR1 and BLR2 or and indirect impact of ENVOY can be assumed. In order to get a first guideline, whether the genes encoding plant cell wall degrading enzymes could be subject to direct regulation by the two photoreceptors or other factors associated to light response, we screened the promotors of genes encoding proteins presumably involved in plant cell wall degradation for the presence of light responsive promotor motifs.

The region within 1000 bp upstream of the ATG codon was searched for specific motifs (Table 1). Gene sequences were obtained from the *Trichoderma reesei*, v2.0 genome database http://genome.jgi-psf.org/Trire2/Trire2.home.html. A complex consisting of *N. crassa *WC-1 and WC-2, the homologues of *T. reesei *BLR1 and BLR2, was reported to bind light-response elements (LREs) with the consensus sequence of GATNC--CGATN, where N can be any nucleotide but the same in both repeats [25]. An LRE motif was only accepted if its length did not exceed 50 bp. As both BLR1 and BLR2 have the characteristic to function as zinc-finger GATA factors, we also screened for the HGATAR (H = C, T, A) consensus sequence [26]. However, binding of these transcription factors to such GATA-sequences was not reported so far. The EUM1 motif which had been identified in the *env1 *as well as the *N. crassa vvd *promotors and thereafter detected in the cellobiohydrolase promotors *cbh1 *(*cel7a*) and *cbh2 *(*cel6a*) [20] was searched because of the light-dependent complex formation detected on EUM1 in both the *env1 *and *gna3 *promotor [23,27]. The proteins constituting the EUM1 binding complex are currently unknown. The analyzed genes were associated with possible activities (Table 1), however, in some cases the lack of knowledge on cellular localization of beta-glucosidases implicates uncertainty as to their involvement in extracellular substrate degradation.

**Table 1:** Results of promoter analysis (1000 bp upstream of ATG) of genes coding cellulolytic enzymes.

Gene	EUM1	GATA	LRE	Enzyme	Activity
*cbh1/cel7a*	-37*r*	-188*f*, -182*f*, -258*f*, -890*f*		CBH	FPA

*cbh2/cel6a*	-159*f*, -1032*r*	-713*r*, -838*f*		CBH	FPA

*egl1/cel7b*		-28*r*, -232*r*, -421*r*		EG	FPA, EGA

*egl2/cel5a*	-168*r*	-25*f*, -807*r*, -1161*f*, -1229*f*		EG	FPA, EGA

*cel5b*		-1186*r*, -1281*r*		EG	FPA, EGA

*egl3/cel12a*	-87*r*	-827*r*		EG	FPA, EGA

*egl4/cel61a*	-752*f*	-727*r*, -1023*r*		EG	FPA, EGA

*cel61b*		-61*f*, -109*r*, -815*f*		EG	FPA, EGA

*egl5/cel45a*		-508*f*, -378*r*		EG	FPA, EGA

*cel74a*	-80*r*, -736*r*, -949*r*			XYL-EG	n.d.

*bgl1/cel3a*	-889*r*, -1122*r*	-548*r*, -1041*f*		EX-BG	FPA, BGA

*cel3b*	-660*r*	-823*f*		BG	FPA^a^, BGA^a^

*cel3c*	-275*f*, -318*r*	-71*r*, -262*r*, -683*f*	-869 to -829*r*	BG	FPA^a^, BGA^a^

*cel3d*		-767*f*, -1222*r*		BG	FPA^a^, BGA^a^

*cel3e*	-772*r*			BG	FPA^a^, BGA^a^

*bgl2/cel1a*		-137*r*, -202*r*, -402*r*, -710*r*		IN-BG	n.d.

*cel1b*	-194*f*, -485*r*	-12*f*, -528*r*	-961 to -941*r*	BG	FPA^a^, BGA^a^

*xyn1*		-986*r*, -868*f*		XYL	XYLA

*xyn2*	-529*f*			XYL	XYLA

*xyn3*	-880*f*	-1092*r*	-77 to -69*r*	XYL	XYLA

*bxl1*		-741*f*, -473*f*		B-XYL	XYLA

^a ^localization of the enzyme not known therefore its contribution to some activities are uncertain

Abbreviations:

CBH - cellobiohydrolase, EG - endo-glucanase, XYL-EG - xyloglucanase, EX-BG - extracellular beta-glucosidase, BG - beta-glucosidase, IN-BG - intracellular beta-glucosidase, XYL - xylanase, B-XYL - beta-xylanase, FPA - Filter Paper Activity, EGA - endoglucanase activity, BGA - beta-glucosidase activity, XYLA - xylanase activity

Location of the respective motif within a promotor is given upstream of ATG, orientation is also indicated (forward, *f *or reverse, *r*).

As presented in Table 1 the EUM1 sequence was found in the promoters of genes for all types of cellulolytic activities. These findings may be interpreted to correlate with the effect of light on cellulase transcription as detected earlier. The highest number of EUM1 motifs is present in the promoter of *cel74a*, which encodes a xyloglucanase.

LRE motifs were only found in the promoters of three genes, two of which encode beta-glucosidases (*cel1b*, *cel3c*), albeit the localization of the encoded proteins is unknown. The third LRE was found in the promoter of a xylanase gene (*xyn3*).

GATA binding sites were found in most promoter regions. Since GATA factors are wide spread regulation elements and due to the high number of motifs found it is hard to predict any relation to light modulated cellulase gene expression, although binding of the photoreceptor complex cannot be excluded. Interestingly, the promoter of *cel5b *encoding an endo-glucanase was the only one where none of these consensus sequences associated with light response was found.

Consequently, while in some cases LRE motifs would render a direct regulation by the photoreceptors BLR1 and BLR2 supposable and several EUM1 motifs suggest binding of (as yet uncharacterized) light regulatory factors, other mechanisms regulating the production of cellulolytic enzymes are likely to contribute to the modulated output in light and darkness.

### Cultivation of mutants in *env1*, *blr1 *and *blr2 *for investigation of cellulase gene expression

In order to obtain preliminary information on whether the transcriptional data on the influence of *env1*, *blr1 *and *blr2 *on cellulase regulation [15,20] would correspond to the cellulolytic efficiency of the enzyme mixture secreted by the respective deletion strains, we first performed shake flask cultures. We found that all three strains showed a significantly higher specific cellulase activity on Mandels-Andreotti medium with 1% (w/v) microcrystalline cellulose than the wild-type strain up to 120 hours of cultivation (data not shown). Considering the transcription data for *cbh1 *in these strains [15] this higher efficiency was surprising. Therefore we chose to use all three strains for analysis of their efficiency in a laboratory scale fermenter and again tested them in shake flask cultures for cellulase expression in the medium to be used for fermentation, which confirmed the results above.

In fermenter cultivations, similar patterns of pH adjustment and oxygen supply were observed for each strain. After approximately 5 hours of lag phase, pO_2 _and pH began to decrease indicating growth of the fungus. The fermentation parameters recorded suggested that the modified strains have no increased oxygen and inorganic nitrogen demand. Fermentations were aborted after 73 hours of run when the oxygen level had reached 50 - 60% and acid addition was detected.

### Enhanced enzyme production by strains with defects in the light response pathway

**Figure 1:**
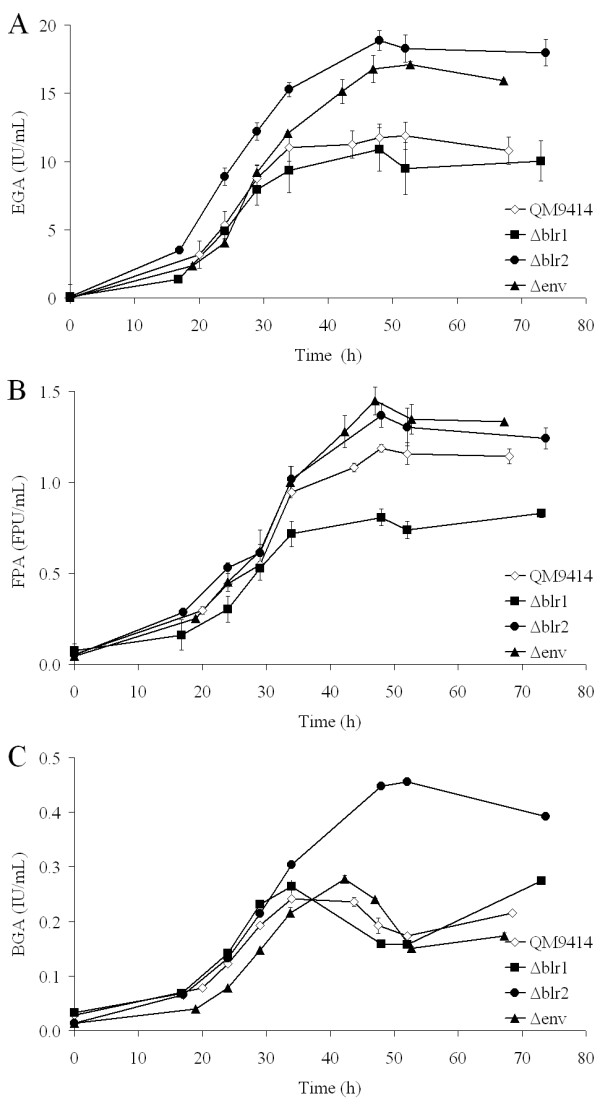
**Endoglucanase (A), FPA (B) and beta-glucosidase (C) activity trends during the fermentation of *T. reesei *QM9414 (empty diamonds), *Δenv1 *(black triangle), *Δblr1 *(black square) and *Δblr2 *(black circle)**. Activities were measured in triplicate and are presented with standard deviation.

The degradative potential of the enzyme mixtures secreted by wild-type and mutant strains was first characterized using dyed Azo-CM-cellulose (Megazyme), which revealed the effectiveness of the whole cellulase enzyme mixture, especially of endo-1,4-β-D-glucanases. (Figure 1A). Peaks of endo-glucanase activity in the culture medium were reached between 48 and 54 hours and after that slightly declined. Highest activity was measured in case of strain *Δblr2 *followed by *Δenv1*, which are 59.0% (p-value 3·10^-6^) and 44.2% (p-value 8.5·10^-5^) higher than the wild-type QM9414, respectively. Between *Δblr1 *and wild type no significant difference was found (p-value 0.33).

Analysis of filter paper activity (FPA), which determines the amount of glucose liberated from cellulose, was performed in order to obtain indications as to the extent of exo-cellulase/cellobiohydrolase activity. Maximal activity values were reached after 48 hours in case of each strain, thereafter activity values slightly decreased which is presumably due to protease activity in the medium (Figure 1B). Highest activities were reached by *Δenv1 *and *Δblr2*, (1.45 and 1.37 FPU mL^-1^), respectively, which are 21.9% (p-value 4.4·10^-5^) and 15.3% (p-value 2.5·10^-4^) higher than those of the parental strain QM9414 (1.218 FPU mL^-1^). *Δblr1 *produced 31.9% (p-value 3·10^-6^) less FPU ml^-1 ^than that observed with QM9414. Hence the increased degradative potential of *Δenv1 *and *Δblr2 *is only in part due to enhanced expression of the cellobiohydrolases, but seems to be mainly caused by an improved overall efficiency of the endoglucanase mixture secreted. Nevertheless, it must be considered that FPA also involves the action of endoglucanases and β-glucosidase and can thus not be seen as exclusive determination of cellobiohydrolase activity.

Since the preferred substrate for yeast needed for the final step of conversion of plant material into ethanol is glucose, we also determined β-glucosidase activity (on pNPG).

Initially, curves had the same slope for each strain, but *Δblr2 *reached an 88.4% (p-value 1.2·10^-5^) higher peak value than the wild-type. Activities of *Δblr1 *and *Δenv1 *were practically equal (p-value 0.29), approximately 10% higher than QM9414 (p-values 3.3·10^-4 ^and 8.5·10^-4^, respectively; Figure 1C). This result is in accordance with the findings described above, although it should be kept in mind that also cell wall bound β-glucosidase [28,29] may contribute to endoglucanase activity and FPA *in vivo*.

Finally, we also assessed the potential of the light response mutant strains to degrade the hemicellulosic part of a given plant material. Highest xylanase activities were observed between 48 and 52 hours of fermentation. Surprisingly, despite its high efficiency on cellulose, *Δblr2 *(101.2 ± 7.5 IU/ml) showed approximately the same xylanase activity as QM9414 (103.8 ± 1.8 IU/ml; p-value 0.52) which may be because of the different regulation pattern of hemicellulolytic enzymes. A comparable effect has already been observed in case of *T*. *atroviride *mutants created by random mutagenesis that possessed enhanced FPA production properties but were deficient in xylanase secretion [30]. Peaks of *Δenv1 *(123.2 ± 2.0 IU/ml) and *Δblr1 *(116.7 ± 5.9 IU/ml) were 18.7% (p-value 2.6·10^-4^) and 12.4% (p-value 6·10^-3^) higher than that of QM9414.

The positive effect of *env1 *gene deletion was consistent with the different activities suggesting that one downstream pathway of the regulatory output of ENVOY are the pathways involved in plant cell wall degradation. The fermentation profiles of the strains show that the function of the regulatory mechanisms causing enhanced cellulase production in the mutant strains becomes most significant after 30 hours of fermentation. While for QM9414 and *Δblr1 *enzyme activities stagnate shortly thereafter (possibly because a critical level of activity for sustaining certain nutrient levels is reached), enzyme production/secretion as reflected by still increasing activities continues in *Δblr2 *and *Δenv1*.

### Specific performance of enzyme mixtures

In order to enable a correlation of the performance of the secreted enzyme mixture (i. e. U/mg of secreted protein) with growth and total protein secretion capacity of the individual strains, we determined biomass and protein content of the culture medium during fermentation (Table 2). Biomass production of strain *Δblr1 *was considerably (37.5% (p-value 0.05) after 24 h and 41.4% (p-value 0.015) after 48 h) higher than that of the wild-type QM9414 throughout the fermentation. In contrast, neither *Δblr2 *nor *Δenv1 *showed a significant difference to the wild type (p-values > 0.1). Biomass specific filter paper activity was the highest for *Δenv1*, followed by *Δblr2*.

**Table 2:** Specific FPA of T. reesei QM9414 and its descendents.

Strain	Hour	QM9414	*Δenv1*	*Δblr1*	*Δblr2*
Biomass,	24	0.164 ± 0.026	0.165 ± 0.010	0.215 ± 0.015	0.159 ± 0.002
	
(mg gluA) mL	48	0.290 ± 0.009	0.259 ± 0.032	0.406 ± 0.036	0.285 ± 0.028

Cell specific FPA	24	2.701 ± 0.429	2.739 ± 0.030	1.409 ± 0.228	3.327 ± 0.176
	
FPU (mg gluA)^-1^	48	4.093 ± 0.162	5.591 ± 0.085	1.990 ± 0.214	4.804 ± 0.232

Cell specific EG	24	32.70 ± 3.61	24.35 ± 0.15	22.69 ± 2.93	55.87 ± 3.94
	
IU (mg gluA)^-1^	48	40.54 ± 1.47	64.80 ± 3.92	26.83 ± 3.88	66.19 ± 2.60

Proteins	24	0.195 ± 0.038	0.181 ± 0.001	0.186 ± 0.040	0.378 ± 0.044
	
mg mL^-1^	48	0.399 ± 0.081	0.494 ± 0.018	0.503 ± 0.038	0.805 ± 0.104

Cellulase efficiency	24	2.271 ± 0.359	2.739 ± 0.028	1.629 ± 0.263	1.399 ± 0.074
	
FPU (mg proteins)^-1^	48	2.975 ± 0.118	2.931 ± 0.045	1.606 ± 0.173	1.701 ± 0.082

EG efficiency	24	27.50 ± 3.04	22.19 ± 0.13	26.23 ± 3.39	23.50 ± 1.66
	
IU (mg proteins)	48	29.47 ± 1.07	33.97 ± 2.05	21.65 ± 3.13	23.43 ± 0.92

Cell specific proteins	24	1.189 ± 0.232	1.097 ± 0.006	0.865 ± 0.186	2.377 ± 0.277
	
mg (mg gluA)^-1^	48	1.376 ± 0.279	1.907 ± 0.069	1.239 ± 0.094	2.825 ± 0.365

Mean values of triplicate measurements are presented including standard deviation.

Both BLR1 and BLR2 have been reported to regulate growth of *Trichoderma atroviride *and *T. reesei *on solid media [15,31], however similar effects of deletion of either of these genes have been observed. Nevertheless biomass formation has not been studied on cellulose containing liquid media in *T*. *reesei*. Our results show that biomass formation of *Δenv1 *is only marginally reduced (p-value 0.11) compared to that of the wild type, which is consistent with the finding that growth is not affected by the deletion in darkness [32].

To illustrate the performance of the cellulase mixture (U/mg of total secreted protein), protein specific endoglucanase activity and FPA was also calculated. The enhanced FPA of *Δblr2 *was in line with the highest protein concentration measured among the strains (Table 2). Considering the biomass formation of this strain, this result indicates that the high cellulolytic activity found in the culture medium of this strain is mainly due to an enhanced protein secretion capacity. An alternative interpretation would be that the higher levels of extracellular proteins observed for this strain are due to autolysis. However, the differences in secretion in terms of proteins secreted per biomass are already detectable for all strains after 24 hours of cultivation, when the fungi are clearly in their production phase and are actively growing (Table 2), which renders this hypothesis unlikely.

Despite the high endoglucanase activity values of *Δenv1*, only slightly increased protein concentration was observed. Therefore, in contrast to *Δblr2*, the enzyme mixture secreted by *Δenv1 *seems to be more efficient than that of the other strains investigated in this study. Since in several promotors of plant cell wall degrading enzymes of *T. reesei *promotor motifs known to be involved in light response in other fungi have been detected (Table 1), light dependent adjustment of the proportions of the respective enzymes could be one way to achive this enhanced efficiency.

For the photoreceptor mutants even decreased endoglucanase and FPA performance (EG activity per mg of secreted protein) was obtained. Interestingly, while the biomass-specific FPA for *Δblr1 *is clearly lower than for all other strains, including wild-type, the protein specific FPA is comparable to *Δblr2*. Consequently, the rather low efficiency of this strain despite its enhanced growth may be due to a decreased protein secretion efficiency caused by deletion of *blr1*.

### Analysis of proteins secreted by mutant strains

**Figure 2:**
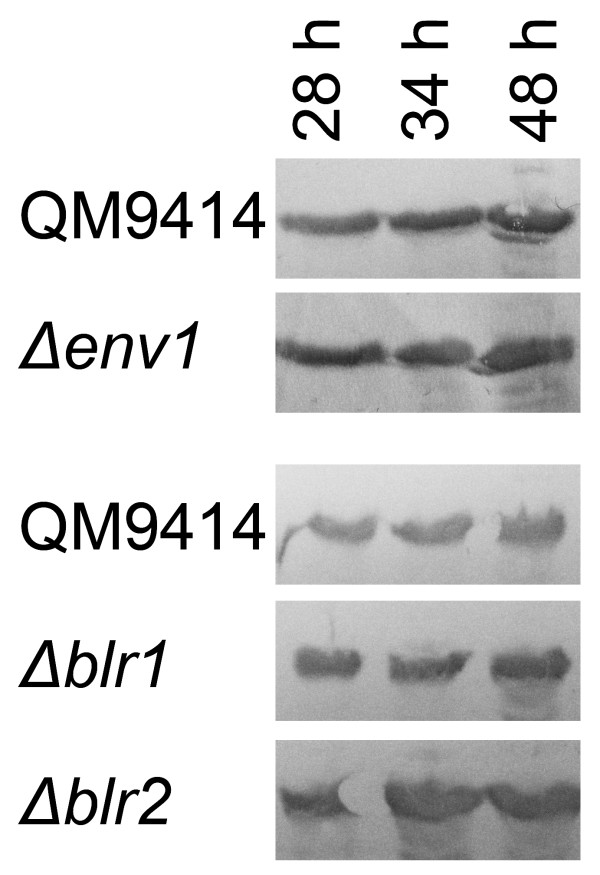
**Western blot showing CBH1/Cel7a abundance in the culture filtrate**. Samples were taken during fermentation at the time points indicated and equal amounts were loaded onto SDS-gels.

In order to analyze whether the increased efficiency is due to an altered expression of the major cellobiohydrolase of *T. reesei *CBH1/Cel7a, Western blotting was performed (Figure 2). In case of Δ*blr2 *clearly higher abundance of CBH1 was observed, indicating that the increased cellulolytic efficiency of this strain is at least in part due to this enzyme. For Δ*env1 *the effect was less clear, suggesting that the improvement of the cellulase mixture found for this strain is likely to be caused by altered regulation of expression of the pool of enzymes contributing to cellulose degradation. Considering the data above, these results correspond well with the enhanced protein secretion capacity of *Δblr2 *and the obviously improved enzyme proportions for *Δenv1*.

## Discussion

Transcriptional responses given to light reflect the changes in the ecological niche that surrounds fungi. *T*. *reesei *as a saprophyte mainly resides in the inside of decaying plant material, that is, in darkness. Presence of light means the open surface of the habitat where the successful dissemination of conidia is expected or existence of possible mating partner is assumed, but also where (energy consuming) measures for protection from harmful UV-light or desiccation have to be taken. The different physiological requirements of growth on the surface or within its substrate are reflected by the metabolic differences between these conditions [10,33], which can be exploited for strain improvement.

One of our most surprising results in this study was the finding that in contrast to transcription of *cbh1/cel7a*, which decreases in deletion mutants of *blr1 *and *blr2 *[15], cellulase activity in the culture medium of the respective strains was nevertheless increased or at least remained at wild-type levels in case of *Δblr1*. Interestingly, comparable effects have been observed for *N. crassa *(M. Schmoll, manuscript in preparation). Hence, this study will be the basis for further research to reveal the molecular basis for this phenomenon. In fact, in accordance with the results presented here, light has previously been reported to influence protein secretion in *N. crassa *[34], however a role of the photoreceptors in this process was not known so far. These results also decreased the applicability of our initial hypothesis that several cellulase genes (other than cbh1/cel7a, which is positively regulated by BLR1 and BLR2 [15]) might be directly regulated by binding of BLR1 and/or BLR2 to their promotors as transcription factors. Nevertheless, at present such a direct regulation cannot be fully ruled out.

The finding of a discrepancy between transcription of the major cellulases and detected cellulase activities in the culture medium is particularly interesting because it suggests that cellulolytic enzymes may not be exclusively regulated on the transcriptional level [35] and references therein), but also posttranscriptionally - presumably in response to light.

Although the signal transduction pathway triggering cellulase gene expression is far from being well-established, first insights are already available. Besides the light response pathway, signals related to sulphur metabolism as well as heterotrimeric G-protein signaling and the cAMP-pathway are involved in regulation of cellulase gene transcription. Addition of the organic sulphur source methionine decreases transcription of *cbh1/cel7a *below detection limits only in light [36]. Deletion of the G-protein alpha subunit GNA1 leads to strongly increased transcription of *cbh1/cel7a *in darkness [24] and constitutive activation of GNA3 causes considerably increased transcription of this gene in light [23]. Hence the relevance of the signals transmitted by GNA1 and GNA3 as well as the sulphur signal must be dependent on the light status, which is perceived and transmitted by the photoreceptors BLR1 and BLR2 [15]. The results presented here could be interpreted in a way that the extent of cellulase transcription is set in response to environmental signals (as transmitted for example by G-proteins via cAMP and phosphorylation to transcription factors), but that the distribution of resources for the energy consuming process of translation and secretion is at least in part governed by the photoreceptors BLR1 and BLR2 and possibly by ENVOY. The fact that these proteins and their orthologues regulate multiple targets [16,32,33] supports this hypothesis. Therefore it will be interesting to learn, whether or not the efficiency of strains engineered for high transcriptional activity of cellulase promotors - such as for example by deletion of *gna1 *- but also strains resulting from random mutagensis, can still be improved by deletion of *blr2*, which might act negatively on secretion. Additionally, elucidation of the mechanism responsible for the impact of components of the light signaling pathways on the secretion machinery warrants further investigations.

It is interesting that different effects have been observed for deletion of *blr1*, *blr2 *and *env1*. On the one hand this finding indicates that BLR1 and BLR2 do not act as a complex under our experimental conditions (mainly darkness) in their regulatory function targeting cellulase gene expression, but have individual functions. On the other hand it also suggests that although induction of *env1 *transcription is abolished upon deletion of *blr1 *or *blr2 *[15], the consequences of deletion of *env1 *are not similar to those of deletion of these photoreceptors. Hence BLR1 and BLR2 do not exert their function via ENV1, which confirms the hypothesis proposed earlier [15].

The increased efficiency of the enzyme mixture secreted by *Δenv1 *further renders a closer investigation of the postulated coregulation of cellulases [35] and references therein] interesting, especially with respect to different light conditions and the regulators involved in transmission of this signal.

The lower efficiency of *Δblr1 *in terms of protein secretion could be due to a counteracting effect of BLR1 compared to BLR2 in secretion. However, in *N. crassa *it has been shown that in darkness the homologue of BLR2, WC-2, is present in excess over WC-1 [37]. Consequently, the effect seen for *Δblr1 *is more likely to be due to an increased availability of BLR2, whose binding partner has been removed resulting in additional BLR2 proteins, now free to exert their negative function on secretion.

Effects of light on metabolic processes have been shown in numerous fungi and for many target mechanisms [10,12]. Therefore the function of the main components of the light perception machinery in regulation of plant cell wall degrading enzymes is not without precedent. A connection between carbon sensing and the function of BLR-1 and BLR-2 has been suggested for *T. atroviride *[31] and different roles in these metabolic functions have been detected: While both proteins are required to adjust the intensity of the response to a certain carbon source, BLR-1 is responsible for carbon source selectivity [38]. For ENV1 a regulatory function in cellulase transcription had been shown in *T. reesei *[20]. Although the most obvious functions of BLR1, BLR2 and ENV1 have been observed and studied in light, it also has been shown clearly that these proteins and their homologues in other fungi additionally have functions in darkness [15,32,33,38]. Hence the investigation of the light response machinery as done in this study, but also of its downstream targets, with respect to industrial fermentations opens up a new strategy for strain improvement aimed at more efficient biofuel production.

## Conclusions

With this study we demonstrated that components of light signaling pathways have an important role also in darkness related to carbon sensing, and highlight that factors which have been yet not associated with industrial cellulase expression still can have impact on it. From a practical point of view these modified strains can be used to enhance productivity, thus lower price of enzyme production which is essential for second generation biofuel breakthrough. Further studies to elucidate the complex signaling and regulatory network through which cellulase transcription and consequently expression can be triggered are already in progress. Our study thus provides new insights into this machinery, which can be exploited to enhance biotechnological fermentation at different stages of regulation in fungi.

## Materials and methods

### Strains

*Trichoderma reesei *QM9414 (ATCC26921) and the following descendents lacking parts of light sensing pathways were used through this study: *Δenv*, *Δblr1 *and *Δblr2*, missing the open reading frames of the respective genes [15]. Strains were maintained on malt agar plates (30 g L^-1 ^malt extract, 1 g L^-1 ^peptone, 20 g L^-1 ^agar) in darkness at 30°C.

### Preculture preparation and shake flask cultures

Conidia from two weeks old plates were harvested with sterile distilled water. An adequate volume of this suspension to reach 10^8 ^conidia L^-1 ^final concentration was transferred to 750 ml Erlenmeyer flasks containing 200 ml of Mandels-Andreotti basal medium [39] prepared in 0.1 M citrate-phosphate buffer (pH 5.0) and supplemented with peptone to a final concentration of 1 g L^-1^. As carbon source, 10 g L^-1 ^Solka Floc 200 (International Fiber Corporation, North Tonawanda, NY, USA) was added. After 3 days of cultivation in constant darkness at 30°C on a rotary shaker (250 rpm) flasks were used to inoculate fermentation medium at 10% (v/v).

### Fermentation

Every strain was analyzed in a 30 L double-walled stainless steel laboratory fermenter (Biostat C-DCU 3, B Braun Biotech, Germany) equipped with pO2, pH, temperature, pressure and foam sensors in 20 L working volume. Agitation was provided by a top-drive agitator with 3 Rushton impellers each with 6 blades.

Medium components (10 g L^-1 ^Solka Floc and 5 g L^-1 ^wheat distiller's grain, 0.83 g L^-1 ^KH_2_PO_4 _and 0.83 g L^-1 ^(NH_4_)_2_SO_4_) were suspended in tap water sterilized in the mounted device and subsequently ten flasks of the precultures were combined aseptically into the sterile inoculating equipment resulting in 2 L of inoculum, which was transferred into the fermentor.

pH was continuously controlled and adjusted to 5.8 by addition of 10% (v/v) NH_4_OH or 10% (v/v) H_3_PO_4_. pO_2 _level was kept on 30% by cascade control of air flow rate as first priority, varying between 5 and 12 L min^-1 ^and velocity of agitation as secondary, shifting between 250 and 600 rpm. Beyond 30% of dissolved oxygen content (DO), air flow rate and agitation worked on the minimal values for both variables. Temperature was set to 28°C. Operation parameters (pH, volume of added base and acid, pO_2_, stirring velocity and air flow rate) were recorded by MFCS/win 2.0 (B Braun Biotech) software. To control foaming, ionic antifoam emulsion (Sigma-Aldrich Antifoam A) was added automatically. Fermentations were carried out in duplicate.

Samples were withdrawn regularly, centrifuged (3400 g, 5 minutes) and supernatants were analyzed for enzyme activities and extracellular protein content. Biomass measurements were carried out daily from fermentation broth (see below).

### Western blot analysis

Proteins from 0.5 ml fermentation supernatant were precipitated by adding 1 ml of 96% ethanol. Western blotting was performed according to standard protocols [40]. Briefly, after centrifugation the pellet was dissolved in 0.2 ml SDS buffer; 10 μl representing equal amounts of culture filtrate were loaded into a 7.5% SDS-PAGE gel and run at 15 mA. Transfer to nitro-cellulose membranes (Hybond-C Extra, Amersham Biosiences, UK) was done by semidry electroblotting and antibodies against the major cellulase CBH1 as well as horseradish peroxidase-conjugated anti-mouse IgG (Promega, Madison, US) were used for analysis of expression of cellulases.

### Analytical assays

For the Filter Paper Activity (FPA) assay 0.5 ml suitably diluted supernatant to liberate approximately 1 mg glucose equivalent was mixed with 1.0 ml of 0.05 M Na-acetate buffer (pH 4.8) and a 6 × 1 cm strip of Whatman grade 1 filter paper was added. After incubation for 1 hour at 50°C, 3 ml of dinitrosalicylic acid reagent [41] was added, kept at 100°C for 5 minutes, diluted with 16 ml of distilled water and reducing sugar content was measured at 550 nm. The filter paper unit (FPU) was defined as the amount of glucose released given in μmol min^-1^.

To measure xylanase activity, 0.1 ml properly diluted supernatant to liberate approximately 0.2 μg xylose was added to the mixture of 0.4 ml 0.05 M citrate buffer (pH 5.3) and 0.5 ml of 1% (w/v) birchwood xylan (Sigma Aldrich) solution prepared the same buffer. After incubation at 50°C for 10 minutes the reaction was terminated by adding 1.5 ml of dinitrosalicylic acid reagent and then the mixture was kept at 100°C for 5 minutes. Absorbance was measured at 550 nm. A xylose calibration curve was used to calculate the activity which was defined as the amount of xylose released given in μmol min^-1^.

β-glucosidase activity was assayed according to the procedure of [42] using 4-nitrophenyl-β-D-glucopyranoside (Sigma-Aldrich) as substrate.

For endo-glucanase activity measurement tenfold diluted supernatant was added to Azo-CM-cellulose solution (S-ACMC; Megazyme International Ltd., Ireland). Procedure was carried out according to the manufacturer's instructions. Extracellular protein content was determined by Coomassie Blue G250 reagent [43] using Bovine Serum Albumin as standard.

All measurements were carried out at least in duplicate.

For biomass determination 100 ml whole fermentation broth was filtered onto a dry and preweighed filter cloth and washed with distilled water. Filtration was carried out in triplicate. After drying at 105°C for 6 hours the filter cloth containing filter cake was weighed again. For further analysis 0.5 g homogenous sample from each filter cake was subjected to a two step sulfuric acid hydrolysis to release N-glucosamine from fungal cell wall: the sample was incubated with 60% (w/v) sulfuric acid at room temperature for 24 hours with subsequent dilution to 1 N and hydrolysis at elevated temperature (121°C for 1 hour) [44]. The cooled mixture was then neutralized by addition of 1 N solution of NaOH and total volume was measured. A portion was centrifuged at 3400 g for 5 minutes and supernatants were analyzed for N-glucosamine by the method of Blix [45] as described by Bussari *et al*. [46]. N-glucosamine content was calculated according to a calibration curve prepared with reagent grade N-glucosamine.HCl (Sigma-Aldrich). Cell mass is expressed as mg N-glucosamine/ml fermentation broth.

Statistical analyses were performed with STATISTICA 8.0 software (StatSoft, Inc., Tulsa, OK, USA). The significance level for t-test for independent samples was set to a p-value of 0.05.

## Competing interests

The authors declare that they have no competing interests.

## Authors' contributions

MGK performed fermentations (together with ZM and GN) and sequence analysis and participated in drafting the manuscript. AS performed Western blot analysis. KR supervised the fermentations. MS conceived of the study, participated in its design and coordination and wrote the final version of the manuscript. All authors read and approved the manuscript.
